# Aging Cell and the growing pains of translational geroscience

**DOI:** 10.1111/acel.13769

**Published:** 2023-01-12

**Authors:** Monty Montano

*Aging Cell*, born in 2002, has contributed to the explosive growth in understanding the biology of aging under the dedicated stewardship of the prior Editors. On behalf of the broad readership and scientific community of *Aging Cell*, let me start by expressing our profound gratitude and recognition of the former Editors‐in‐Chief, editors, and editorial staff for their vision and dedication to advancing aging research. Enabled by model systems, many of which are evolutionarily conserved, *Aging Cell* has served as a forum for communicating many of the key advances in the biology of aging, including (i) efforts to define aging‐related phenotypes arising from primary pathways (i.e., hallmarks or pillars), (ii) omics‐driven efforts to develop composite tools that measure the pace of aging (e.g., molecular clocks), and (iii) identify therapeutic compounds that may tip the balance between gerodrivers in favor of geroprotectors.

These collective advances in the molecular details of the aging process are now poised to be translated to mitigate or reverse age‐related conditions at the whole person level, as well as to extend lifespan and optimize healthspan. The looming prospect of an increasing prevalence of chronic unwellness with aging challenges us at *Aging Cell* to recognize and prioritize gaps in the translation of advances in geroscience.

As the new Editor‐in‐Chief, I am deeply honored to take the helm of *Aging Cell* and committed to positioning *Aging Cell* as the leading journal in basic and translational geroscience. Below, I articulate my vision for *Aging Cell*, starting with a fundamental question: *Why should you consider your very best work for Aging Cell?*

*Aging Cell* will continue its rich tradition of focusing on rigorous studies that (i) convey novel mechanisms that, ideally in humans, predict and/or strongly associate with the pillars of aging; (ii) define the interaction between aging hallmarks and comorbid conditions; (iii) identify molecular determinants of physical and cognitive function that contribute to resilience and healthy aging; and (iv) improve our understanding on how the aging process relates to the evolving nature of immune, metabolic, and infectious as well as microbiome parameters across the lifespan.

To further position *Aging Cell* as the premier home for aging research, the editorial board is committed to give their full consideration to broadening the scope of *Aging Cell* to include:

1. Rigorously conducted basic, translational, and/or clinical studies that support “out‐of‐the‐box” new theories and conceptual advances.
2. “Big data” studies that, by synthesizing extant publicly available datasets (e.g., genetic, gene expression, transcriptome, proteome, methylome), bring to the fore fresh ideas and revisitation of embedded ideas.
3. Comparative lab‐based studies and field studies in real‐world settings.
4. Advancements in the field of in vivo and sensor‐based monitoring of the aging process.
5. The challenge of deconvoluting the heterogeneity of the aging process across the arc of the lifespan, including crosstalk between organ systems and asynchronous decline in organ function.
6. Non‐human models that inform patient‐defined health outcomes.
7. Rigorously designed phase I/II clinical trials of geroscience‐informed interventions.

We all complain about the review process. We are mindful that no system is perfect. Personal bias and preconceived notions creep into the review. While we cannot fix the system, at *Aging Cell*, we are committed to the following considerations.

1. Rapid and objective reviews.
2. Discordant reviews of studies judged meritorious with high standards will be resolved by engaging the editorial board and the reviewers directly.
3. Author engagement and dialog to build an *Aging Cell* community of scholars.

In addition to the aforementioned, the following initiatives have been planned.

1. “Perspective” viewpoints focused on advances in the biology and theory of aging from established and emerging key thought leaders.
2. Special issues focusing on interdisciplinary studies that bridge aging sciences with other life science disciplines (e.g., cancer, neuroscience, immunology, bioengineering, tissue regeneration, and repair).
3. Summaries of key meeting/symposia proceedings and web interviews with key opinion leaders.
4. Community outreach and engagement to catalyze discourse on the biology of aging across academia, industry, and public health domains.

I hope that I have conveyed an ambitious agenda for *Aging Cell* that will set it apart from other journals and why I am encouraging investigators across the aging field to consider their very best work for this journal. I see a bright future for aging research and this journal. I obviously cannot actualize this agenda alone. I am revamping and expanding our editorial board, as well as recruiting early‐stage researchers working on emerging areas of basic and translational geroscience that are likely to impact healthspan worldwide.

Let me close by saying that the aging field is at an inflection point, shifting from nascent knowledge to translating this knowledge to extend lifespan and improve healthspan. *Aging Cell* is committed to making this inflection a success. I recognize that there will be growing pains, but perhaps, this is inevitable to progress that I hope we can experience together as a community focused on improving the human condition through our collective efforts.

